# A Meta-analysis of Transanal Endoscopic Microsurgery versus Total Mesorectal Excision in the Treatment of Rectal Cancer

**DOI:** 10.1055/s-0041-1735587

**Published:** 2021-09-14

**Authors:** Nasir Zaheer Ahmad, Muhammad Hasan Abbas, Mohamed H. Abunada, Amjad Parvaiz

**Affiliations:** 1 Department of Surgery, University Hospital Limerick, Limerick, Republic of Ireland; 2 Department of Surgery, Russells Hall Hospital, NHS Trust, West Midlands, Dudley, United Kingdom; 3 Department of Surgery, Hamad Medical Hospital, Doha, Qatar; 4 Faculty of Health Sciences, University of Portsmouth, Portsmouth, England; 5 Department of Colorectal Surgery, Poole NHS Trust, Poole, United Kingdom

**Keywords:** rectal cancer, microsurgery, total mesorectal excision

## Abstract

**Background** Transanal endoscopic microsurgery (TEMS) has been suggested as an alternative to total mesorectal excision (TME) in the treatment of early rectal cancers. The extended role of TEMS for higher stage rectal cancers after neoadjuvant therapy is also experimented. The aim of this meta-analysis was to compare the oncological outcomes and report on the evidence-based clinical supremacy of either technique.

**Methods** Medline, Embase, and Cochrane databases were searched for the randomized controlled trials comparing the oncological and perioperative outcomes of TEMS and a radical TME. A local recurrence and postoperative complications were analyzed as primary end points. Intraoperative blood loss, operation time, and duration of hospital stay were compared as secondary end points.

**Results** There was no statistical difference in the local recurrence or postoperative complications with a risk ratio of 1.898 and 0.753 and *p* -values of 0.296 and 0.306, respectively, for TEMS and TME. A marked statistical significance in favor of TEMS was observed for secondary end points. There was standard difference in means of −4.697, −6.940, and −5.685 with *p* -values of 0.001, 0.005, and 0.001 for blood loss, operation time, and hospital stay, respectively.

**Conclusion** TEMS procedure is a viable alternative to TME in the treatment of early rectal cancers. An extended role of TEMS after neoadjuvant therapy may also be offered to a selected group of patients. TME surgery remains the standard of care in more advanced rectal cancers.

Rectal cancer surgery was historically associated with a high local recurrence rate until the emergence of total mesorectal excision (TME) in 1982. 1 The concept of TME has been accepted widely and is now considered to be a gold standard in the surgical treatment of rectal cancer. A radical surgery for rectal cancer in the form of a TME is often associated with the risk of serious perioperative morbidity and mortality and has led to a search for less aggressive alternatives particularly in patients who are unfit and have significant comorbidities.

A transanal endoscopic microsurgery (TEMS) was introduced in 1983 by Buess et al as an alternative to the existing transanal excision techniques used for resection of rectal adenomas. 2 3 The issues with the traditional transanal excisions were difficult histological interpretation of the surgical specimen because of the operative fragmentation, a high rate of positive margins and especially the inability to access high lying rectal lesions. Early results of TEMS confirmed its ability to excise large circumferential lesions and the lesions as high as 25 cm with precision and safety. 4 5 The superiority of TEMS over transanal excisions was accepted and reported in subsequent observational studies and meta-analysis. 6 7 The indications of TEMS have evolved over the years and consist of a potential role in dilating the colorectal anastomotic strictures, 8 repair of rectovaginal fistulas, 9 transanal rectal prolapse surgery, 10 and its use as a platform for NOTES (Natural Orifice Transluminal Endoscopic Surgery) procedure. 11

TEMS was also proposed as an alternative to TME in the treatment of early rectal cancer and in situations where a radical surgery would carry a significantly higher risk of complications. There have been experimental attempts to extend the indications of a TEMS to even more advanced rectal cancers by utilizing adjuvant therapies. Several randomized controlled trials (RCTs), observational studies, and meta-analysis have compared the results of TEMS with radical TME in dealing with rectal cancer. 12 13 14 15 16 A more recent meta-analysis of RCTs comparing the oncological and short-term outcomes of the two techniques did not find any significant difference in the local recurrence rate between the two techniques. 17 The current meta-analysis of the RCTs and a literature review was conducted to compare the short- and long-term outcomes of TEMS and a radical TME in the treatment of early rectal cancers which could prove useful for clinical decision-making for practicing colorectal surgeons.

## Methods

A literature search of Medline, Embase, and Cochrane databases was performed using the keywords, “transanal endoscopic microsurgery,” OR “total mesorectal excision” AND “rectal cancer.” The search was limited to the RCTs. No language or time constraint was applied to the search strategy. Further manual searching was performed of the references for missing studies. All the titles and selected abstracts were reviewed by two authors. Duplicate studies and irrelevant articles were excluded. Full-text articles of more pertinent publications were retrieved and final decisions to include or exclude a study were made with consensus. RCTs comparing the oncological and perioperative outcomes after TEMS or a radical TME were considered suitable for meta-analysis. In cases of more than one publications by the same authors, only the most recent trial was included in the analysis. Data on patient characteristics, study designs, outcomes, and follow-ups were extracted by one of the authors and counterchecked by the second author. Any discrepancy or disagreement was resolved by input from the senior author. Preferred Reporting Items for Systematic Reviews and Meta-Analyses guidelines were followed for the literature search. 18

### Quality Assessment

The quality of included RCTs was assessed using Jadad scoring system. 19 The assessment was performed across different variables to check for randomization, method of randomization, blinding, and description of follow-ups or the dropouts. None of the RCTs was reported as blinded probably because of the nature of the intervention and scored zero in this area of assessment. RCTs meeting all the criteria of randomization, blinding, and follow-up would have a maximum score of 5. A score of less than 2 would be considered low quality and more than 2 a high quality.

### End Points

The primary end point of local recurrence rate after primary excision was analyzed as a long-term outcome and the postoperative complications related to both techniques were compared as short-term outcomes. Other perioperative outcomes including the hospital stay, operation time, and intraoperative bleeding were also analyzed as secondary end points.

### Statistics

The data from the included RCTs were pooled on the Microsoft Excel. The dichotomous and continuous data were separated for analysis. Heterogeneity among the studies was checked for the primary and secondary outcomes. In case of a significant heterogeneity (a value of <0.1), a random effect model was used for meta-analysis and vice versa. The risk ratio (RR) and 95% confidence interval (CI) was calculated for the dichotomous data and standard difference in means (SDM) along with 95% CI was calculated for the continuous variables. The mean and standard deviation values were estimated using the formulas given by Hozo et al. 20 A publication bias was checked and a sensitivity analysis was done. Comprehensive Meta-Analysis, Version 2 was used for statistics.

## Results

**Fig. 1:**
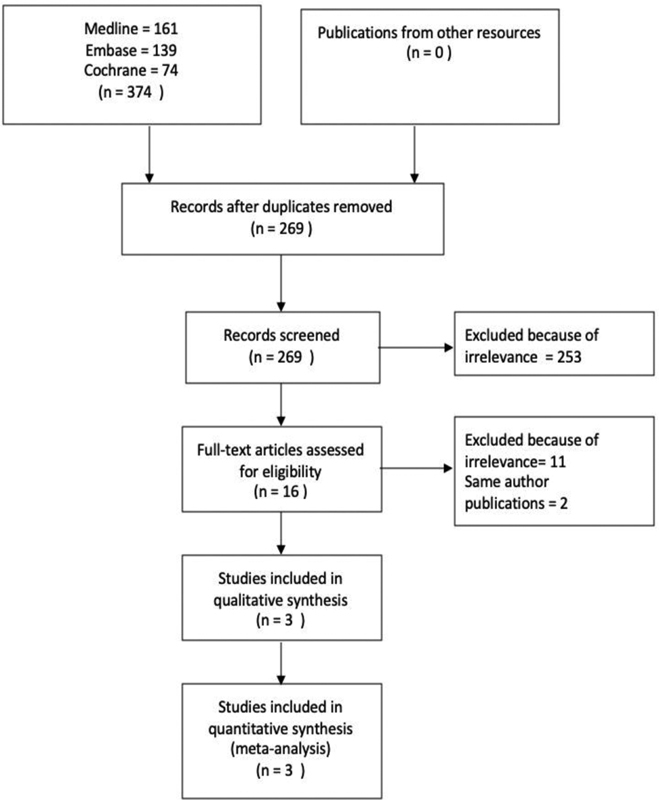
Preferred Reporting Items for Systematic Reviews and Meta-Analyses flow chart.

An advanced literature search of Medline, Embase, and Cochrane databases revealed 161, 139 and 74 publications, respectively. After the exclusions of duplicates and other irrelevant publications, three RCTs were found suitable for meta-analysis 21 22 23 ( Fig. 1 ).

A total number of 208 patients with an average age of 64.6 years in the TEMS group and 64.36 years in the TME group were analyzed according to the study protocols. The rest of the study characteristics are given in Tables 1 and 2 .

**Table 1:** Study characteristics

RCT	Winde et al	Lezoche et al	Chen et al
Year	1996	2012	2013
Country	Germany	Italy	China
Ethical approval	NG	Yes	Yes
Preoperative investigations	Proctoscopy/biopsies, colonoscopy, ERUS, biochemical profile	ERUS, rigid sigmoidoscopy, CT, MRI, colonoscopy and biopsies, tattoo	Rectoscopy, ERUS, CT, MRI
Randomization	Number table	Sealed envelopes	Equal randomization
Power calculation	Not done	Yes	Not done
Jadad scale	3	3	2
Cancer stage	T1	T2	T1, T2
Tumor location	8–18 cm	6 cm	6–15 cm
Tumor size	NG	<3 cm	<3 cm
Preoperative histology	Adenocarcinoma (G1/2)	Well or moderately well-differentiated carcinoma	Moderate or highly differentiated carcinoma
Postoperative histology	Not reported	Not reported	Reported
Inclusion	Adenocarcinoma G1 and G2 T1 tumors	ASA I and II, within 6 cm of anal verge, well (G1) or moderately (G2) differentiated, less than 3 cm diameter	No previous lower abdominal or pelvic surgery Acceptable physical status
Exclusions	Poorly differentiated tumors, higher than T1 stage	ASA III and IV, more proximal tumor, poorly (G3) or undifferentiated (G4) tumors, tumors with lymphovascular or perineural invasion, suspicious lymph node status	Deep tumor invasion Distant metastasis
Confounders	NG	NG	Hypertension, DM, cardiovascular disease
CRT	None	All	None
Bowel preparation	Yes	Yes	NG
Number of surgeons	3	2	NG
Antibiotics	Yes	Yes	NG
Patients	50	100	60
TEMS resection margin	1 cm	NG	0.5–1 cm
TME resection margin	2 cm	NG	2 cm
Frozen section (TEMS/TME)	None	None	Yes
Ileostomy	NG	23 (11 temporary 12 permanent after APR)	9
Rectal perforations	1	None	2
Conversion from TEMS to TME	0	0	2
Violation of study protocol	NG	6	NG
Access	Open	Laparoscopic	Laparoscopic
Drain	Yes	Yes	NG
Investigations at follow-up	Proctoscopy, tumor markers, clinical examination, CXR, ERUS every 3 mo for 2 y and then biannually for 3 y. Annual after 5 y	Tumor markers, clinical examination, sigmoidoscopy 3 monthly for 3 y, then 6 monthly, CT/MRI biannually for 5 y	Tumor markers, USG, CXR biannually CT/MRI, colonoscopy annually
End points	Local and distant recurrences, complications, hospital stay, blood loss, operation time, analgesia requirements, survival rate, and mortality	Local and systemic recurrences, operation time, blood loss, analgesic use, morbidity, hospital stay, 30-d mortality	Operative time, blood loss, recovery time, morbidity, mortality, local recurrence, distant recurrence

Abbreviations: APR, abdominoperineal resection; ASA, American Society of Anesthesiologists; CRT, chemoradiotherapy; CT, computed tomography; CXR, chest X-ray; ERUS, endorectal ultrasound; MRI, magnetic resonance imaging; NG, not given; RCT, randomized controlled trial; TEMS, transanal endoscopic microsurgery; TME, total mesorectal excision.

**Table 2:** Study characteristics

RCT	Winde et al		Lezoche et al		Chen et al	
	TEMS	TME	TEMS	TME	TEMS	TME
Number of patients	24	26	50	50	30	30
Average age	63.7	60.9	60 ± 3	66 ± 2.25	68.8 ± 5.3	66.2 ± 7.7
Secondary operations	2	3	1	3	0	0
Salvage surgery	1	0	NG	NG	1	0
BMI	NG	NG	NG	NG	20.0 ± 0.3	20.1 ± 0.3
Conversions	0	0	0	5	2	0
Major bleeding	1	0	0	10	0	1
Positive margin	NG	NG	0	0	0	0
Margins	1 cm	2 cm	1 cm	NG	0.5–1 cm	2 cm
Lymph nodes retrieved	NG	NG	1	11	NG	NG
Follow-up	40.9 ± 24.6	45.8 ± 24.6	9.6 ± 1.72 Y	9.6 ± 1.9 Y	18.0 ± 2.6	17.5 ± 2.2
Mortality	1	1	10	7	0	0
Tumor distance	NG	NG	4.92 (3–6)	5 (3–6)	7.8 ± 1.6	8.1 ± 1.3
Tumor size	NG	NG	NG	NG	2.3 ± 0.5	2.8 ± 0.6
T1	24	26	0	0	24/30	22/30
T2	0	0	50	50	6/30	8/30
T stage under estimation	Not reported	Not reported	Not reported	Not reported	2	0
Lymphovascular invasion on postoperative specimen	NG	NG	NG	NG	4	7
Adjuvant chemotherapy	NG	NG	0	0	1	8

Abbreviations: BMI, body mass index; NG, not given; RCT, randomized controlled trial; TEMS, transanal endoscopic microsurgery; TME, total mesorectal excision.

**Fig. 2:**
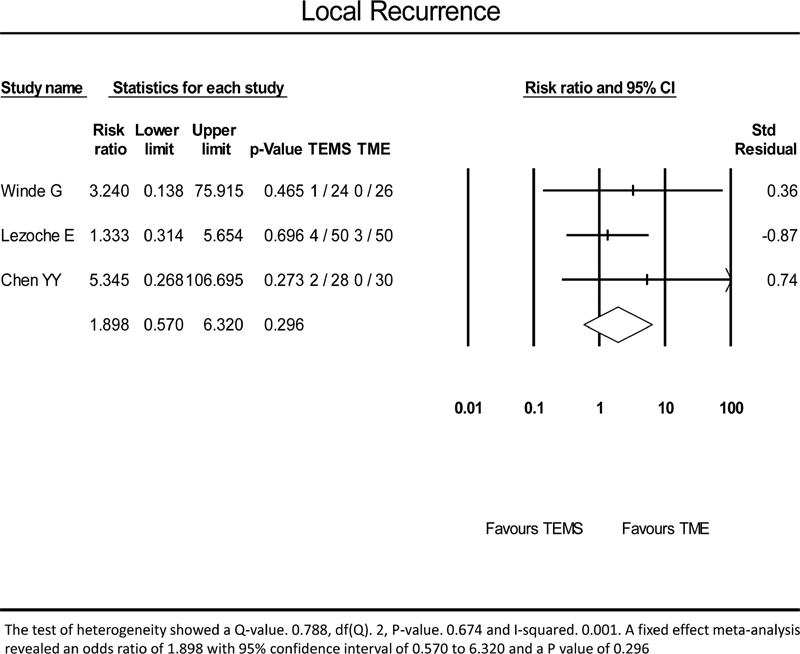
A meta-analysis of local recurrence. TEMS, transanal endoscopic microsurgery; TME, total mesorectal excision.

Local recurrence was seen in 7/103 (6.7%) cases in the TEMS group and 3/105 (2.8%) in the group after radical TME. There was a significant difference in the follow-up in different trials that explains recurrence of more cases in a trial with longest follow-up. A meta-analysis of local recurrence after rectal cancer surgery using the fixed effect model did not show any statistical difference between the two groups with an RR of 1.898 and a *p* -value of 0.296 ( Fig. 2 ).

**Fig. 3:**
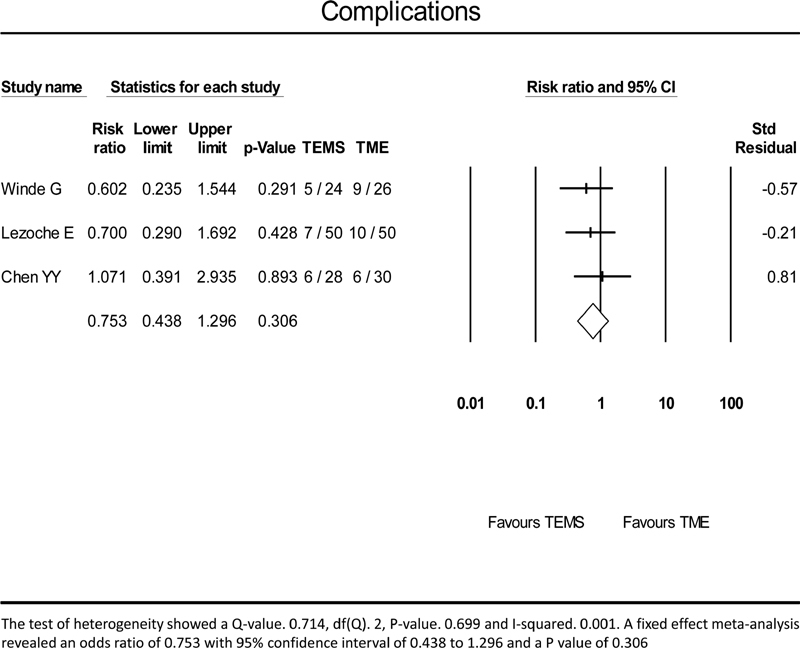
A meta-analysis of complications. TEMS, transanal endoscopic microsurgery; TME, total mesorectal excision.

Postoperative complication rate was 18/103 (17.47%) and 25/105 (23.8%) in the TEMS and the TME groups, respectively. A meta-analysis using the fixed effect model did not show any significant difference between the two groups with an RR of 0.753 and *p* -value of 0.306 ( Fig. 3 ).

As reported in the literature previously, the hospital stay after the surgery was shorter in patients who underwent a TEMS procedure as compared with the patients who had a radical surgery. The average hospital stay was 6.2 and 13 days for the TEMS and TME groups, respectively. There was significant heterogeneity among the studies and a random effect meta-analysis confirmed a significant difference between the two groups in favor of TEMS. The operating time was much shorter for a TEMS procedure as compared with a radical resection, 107.76 and 173.9 minutes, respectively. There was a significant heterogeneity among the studies and a random effect model showed a significant difference in favor of TEMS. The average intraoperative blood loss was 76.23 mL in the TEMS group and 346.23 mL in the TME group. This significant difference is explained by the major bleeds that happened in 11 cases in the radical surgery group only. There was no major bleed in the TEMS group. A random effect meta-analysis favored TEMS and confirmed a significant difference between the two techniques ( Table 3 ).

**Table 3:** A meta-analysis of the hospital stay, operation time, and blood loss

Outcome	SDM	95% confidence interval				Heterogeneity			Favors
		Lower	Upper	SE	*p* -Value	*Q* -Value	*p* -Value	*I* ^2^	
Hospital stay	−5.685	−8.131	−3.239	1.248	0.001	31.387	0.001	93.628	TEMS
Operation time	−6.940	−11.793	−2.087	2.476	0.005	129.71	0.001	98.458	TEMS
Blood loss	−4.697	−7.348	−2.046	1.353	0.001	61.305	0.001	96.738	TEMS

Abbreviations: SDM, standard difference in means; SE, standard error; TEMS, transanal endoscopic microsurgery.

### Publication Bias

**Fig. 4:**
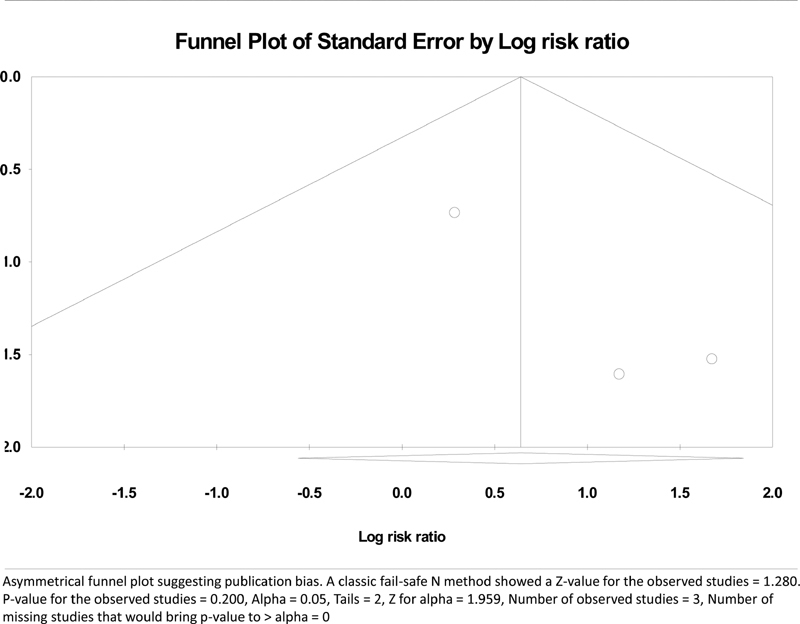
Funnel plot of publication bias.

A publication bias was checked for the primary end point of local recurrence. The funnel plot of the standard error by log odds ratio and 95% CI was asymmetrical suggesting a high possibility of publication bias ( Fig. 4 ). A classic fail-safe N method confirmed the bias and it was established that as the difference between two methods was not significant, no more studies would be required to bring the *p* -value to >0.05. A sensitivity analysis did not have any effect on the results.

## Discussion

TEMS was proposed to treat benign adenomas, not accessible with the conventional instruments for transanal local excision. A high rate of incidental cancers in the postoperative specimens led the surgeons to extend the indications of TEMS for early rectal cancer with anticipated lower risk of recurrence. 24 Low-risk cancers defined as those limited to the submucosa (T1), less than 3 cm in size, exhibit well to moderately differentiated morphology, are Sm1, Haggitt 1–3 and do not show any lymphovascular invasion. 25 The RCTs included in this meta-analysis adapted extensive investigations for accurate preoperative staging of rectal cancers.

The preoperative investigations commonly employed to characterize a rectal cancer include digital rectal examination, rectoscopic biopsies, endorectal ultrasound, magnetic resonance imaging (MRI), and computed tomography of thorax, abdomen, and pelvis to exclude distant metastasis. Endorectal ultrasound and MRI are considered sensitive and diagnostic for the depth of tumor invasion and assessment of regional lymph nodes. However, in a vast majority of the cases, the accurate stage of the tumor could only be determined after the final histology of the excisional specimen. 26 A discrepancy in the preoperative and postoperative histological diagnoses is not uncommon. As a result of this inconsistency, some low-risk early rectal cancers may get operated with radical resections 27 and vice versa. Postoperative pathological stage and a comparison with the preoperative stage were only reported in one of the trials included in this meta-analysis. 23

The outcomes of TEMS depend on proper patient selection which does include selection of a suitable cancer as well. A suitable patient with unsuitable cancer or a suitable cancer with an unsuitable patient are the possible clinical scenarios which would have impact on the outcomes. A radical TME on the other hand does not suffer from this constraint and almost every rectal cancer can be subjected to this type of operation. A careful selection of the cases has been emphasized for optimal outcomes when TEMS is attempted with a curative intent. 28 But even a careful selection of the cases may still lead to less than satisfactory results and a radical resection in the form of a TME may still be necessary in ∼30% of the cases after initial TEMS. 29

A completely correct preoperative staging and even a confirmed diagnosis of low-risk early rectal cancer would still not be enough to eliminate the risks of local recurrence and other complications after TEMS. The importance of a careful patient selection for TEMS cannot be overemphasized as salvage procedures would become necessary to treat the recurrences of TEMS. 30 A low-risk T1 rectal cancer may already have involved the regional lymph nodes that would lead to a high recurrence rate if the lymphatic basin is left untreated. 31 Patients with T1 rectal cancer from the Dutch TME trial when compared with TEMS and TME showed 24% recurrence rate after TEMS and 80% of these patients eventually required radical surgery for the local recurrence. 32

There is a substantial risk of local recurrence especially for large rectal cancers with unfavorable histology. Further treatment of local recurrence depends on the intention to cure or palliate. A TME after the recurrence of TEMS is known to have reasonably good results comparable to primary TME; however, the presence of distant metastasis or the complexity of a salvage procedure would generally have poor prognosis. 33 34 35 36 37 38 39 A recurrence after TEMS may require an abdominoperineal resection (APR) as the salvage procedure and in case of the recurrence involving other pelvic organs, it may become necessary to undertake a pelvic exenteration in which case the outcomes are even poorer. 40 The disastrous outcomes of a second salvage surgery highlight the importance of careful selection of cases for the primary TEMS. 41 The outcomes of the salvage resections are not detailed in the studies included in this meta-analysis.

The role of neoadjuvant chemoradiotherapy (CRT) before undertaking TEMS has been reported with promising results even for locally advanced cancers. 22 42 43 44 On the other hand, it has been established that a local recurrence is more likely to happen after local excision of T2 or T3 rectal cancers. 45 The CART study (Transanal Endoscopic Microsurgery After Radiochemotherapy for Rectal Cancer) confirmed similar results and reported that about one-third of patients after CRT and TEMS would still require radical resection. 46 A relatively higher recurrence rate when operated by TEMS after neoadjuvant therapy was reported by one of the RCTs included in this meta-analysis. A 50% downstaging and downsizing after neoadjuvant therapy is believed to be a prerequisite for TEMS in these cancers and is believed to minimize the risk of recurrence. 22

The risk of complications other than a local recurrence is not different after radical surgery or TEMS. The spectrum of complications includes immediate postoperative issues such as bleeding, anastomotic breakdown, infection, incontinence or rectal pain, and the functional outcomes impacting on quality of life. In addition, a radical surgery may be associated with genitourinary dysfunction and patient may also suffer from the sequelae of an anastomotic leak. Low anterior resection syndrome (LARS), once thought to be a complication, exclusively related to a radical resection for a very low rectal cancer is not entirely true as patients undergoing TEMS have also been reported to suffer from this complication. 46 47 The quality of life disruption after LARS seems to be transient as a comparison of functional outcomes after TEMS or TME for T1 rectal cancer revealed a complete recovery in both groups at 1 year. 48 It has been established that a preoperative CRT and more distal lesions lead to more issues with functional outcomes, but fortunately, these complications are usually self-limiting. 49 The trials included in this meta-analysis did report on rectal pain and anal incontinence, but the occurrence of LARS as a long-term complication was not reported in any of the publications.

This meta-analysis suffers from some inherent limitations which may have an impact on overall effect size calculations. These include limited number of RCTs with a small number of patients, a significant heterogeneity among the studies, inconsistent inclusion and exclusion criteria, diverse protocols for adjuvant and neoadjuvant therapies, and a different duration of follow-ups. Despite these shortcomings, the meta-analysis of primary end points completely rejected the theoretical assumptions of a higher risk of surgical complications after radical surgery and a higher risk of local recurrence rate after TEMS. There was a difference in the surgical approach across the included studies as Winde et al 21 used an open approach for radical resections, whereas laparoscopic resections were performed in the other two trials. 22 23 A distinct statistical difference in favor of TEMS was observed in secondary end points. A shorter hospital stay and duration of surgery would have an impact on the cost effectiveness of the procedure. Similarly, less blood loss would lead to avoidance of perioperative blood transfusions which is considered relevant in cancer surgery.

## Conclusion

There is no convincing evidence that TEMS is superior to TME in terms of the oncological outcomes, but the organ preservation philosophy sounds promising in the treatment of early rectal cancers, and therefore, it should be offered carefully to very selected patients. 50 The extended indications of TEMS in dealing with T2 tumors after neoadjuvant therapy seem somewhat presumptuous, as a similar T stage for radical surgery would go straight for surgery without any neoadjuvant therapy avoiding the hazards of CRT. The argument of a palliative TEMS in patients not fit for a radical surgery seems justified in selected cases but with the evolving concept of wait and watch after CRT, a vigorous surveillance may be another option for these patients. This concept would need more studies to compare the outcomes of two modalities in dealing with these cancers. TEMS definitely has a vital role in the surgical practice but because of the risks of unfavorable outcomes so far in the curative treatment of rectal cancer, this therapeutic modality may be limited to the clinically, radiologically, and histologically proven early rectal cancers.
